# Supplementary material for: Basis of executive functions in fine-grained architecture of cortical and subcortical human brain networks
Source: Cereb Cortex. 2024 Jan 18;34(2):bhad537. doi: 10.1093/cercor/bhad537 (PMC10839840; doi:10.1093/cercor/bhad537)
Supplement: Supplementary_figures_ExecFunc_Assem_et_al_CerCor_bhad537 [file supplementary_figures_execfunc_assem_et_al_cercor_bhad537.pdf]

Supplementary Figures

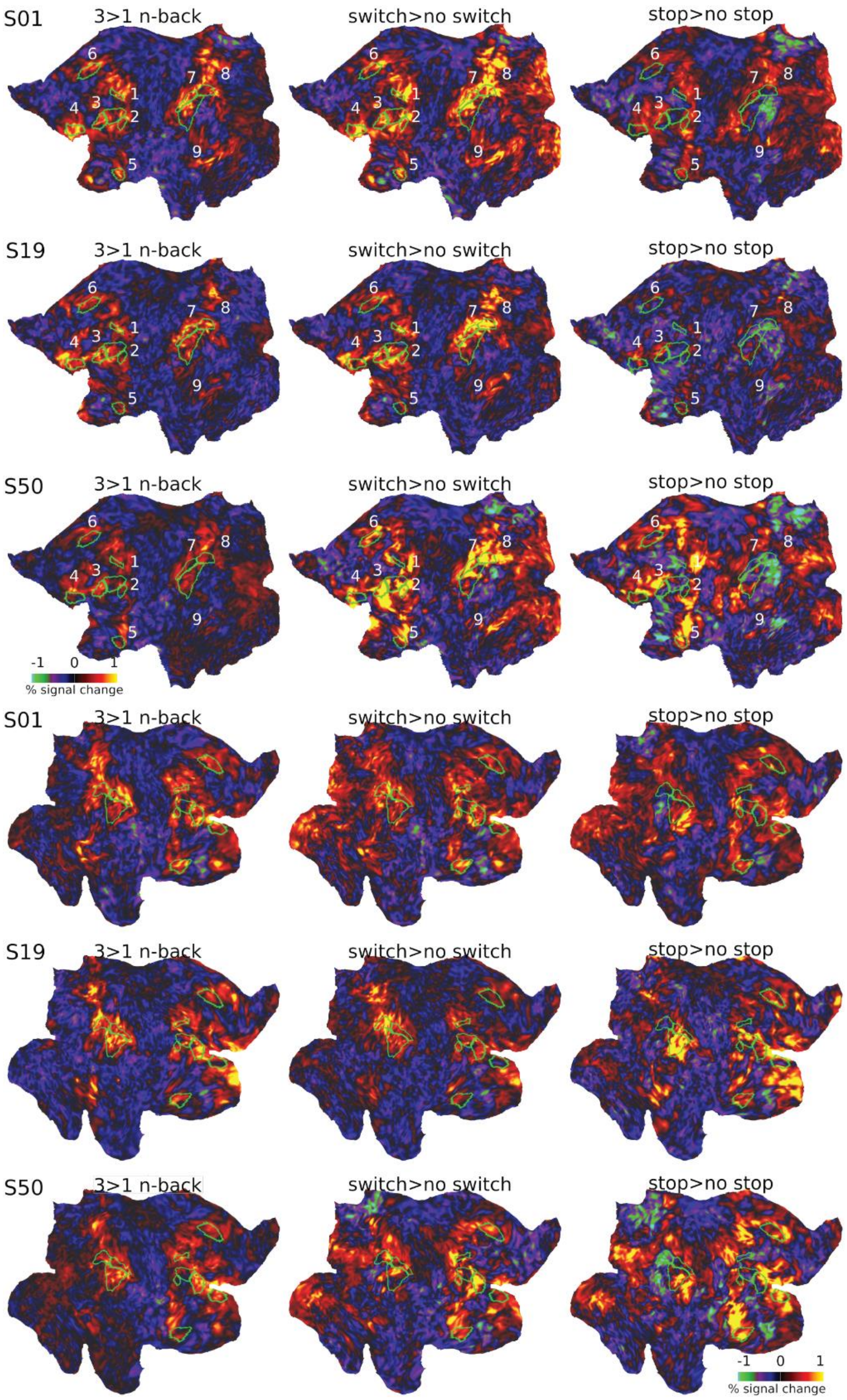

Supplementary Figure 1. Activation maps for each task for three example subjects. Top three rows are left hemisphere. Bottom three rows are the right hemisphere. Core MD borders are outlined in green. Data available at: http://balsa.wustl.edu/Nkm05

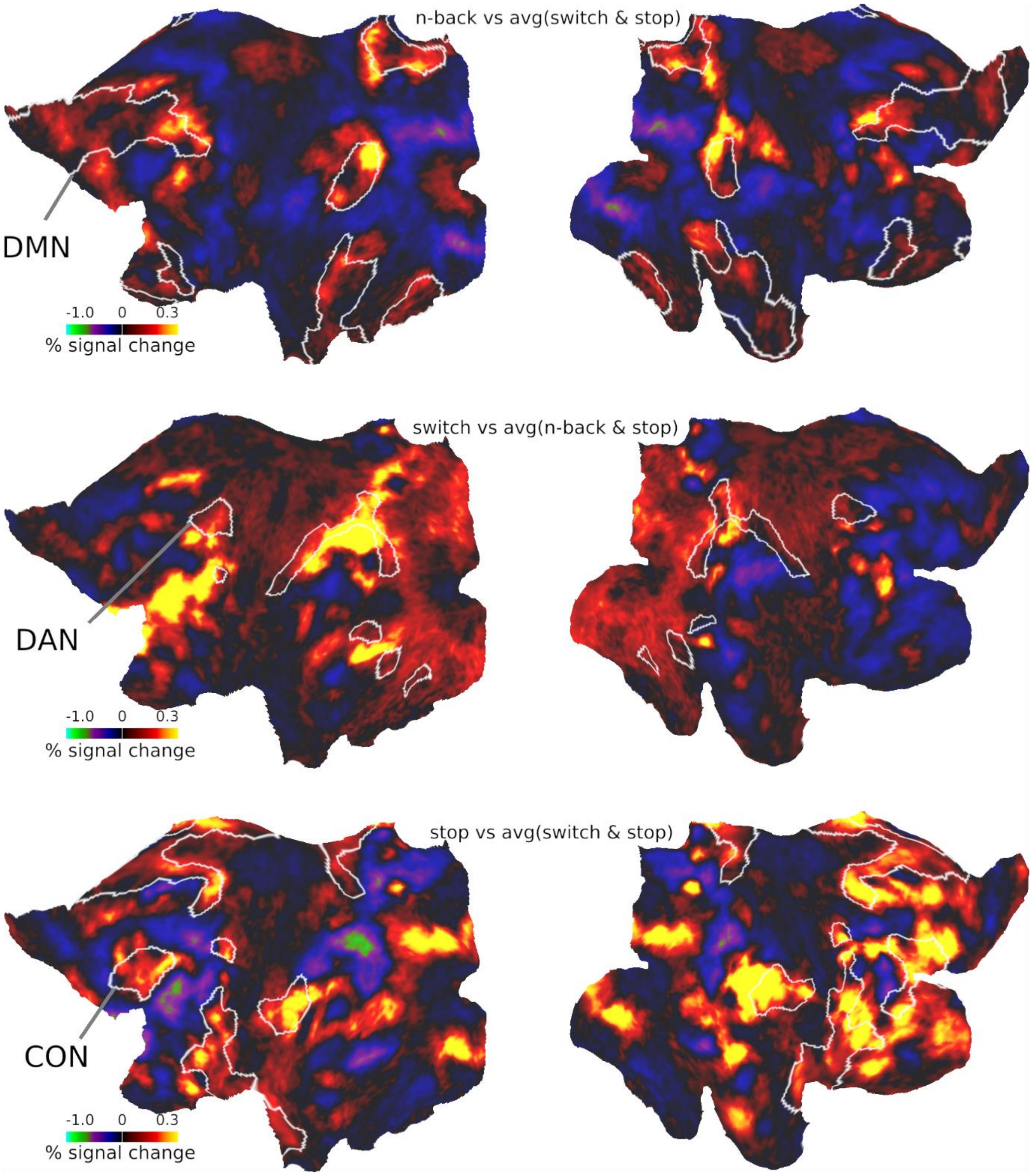

Supplementary Figure 2. Group average activations of each executive task minus the average of the two other tasks. RSN borders are outlined in white. Data available at: http://balsa.wustl.edu/l7kN9

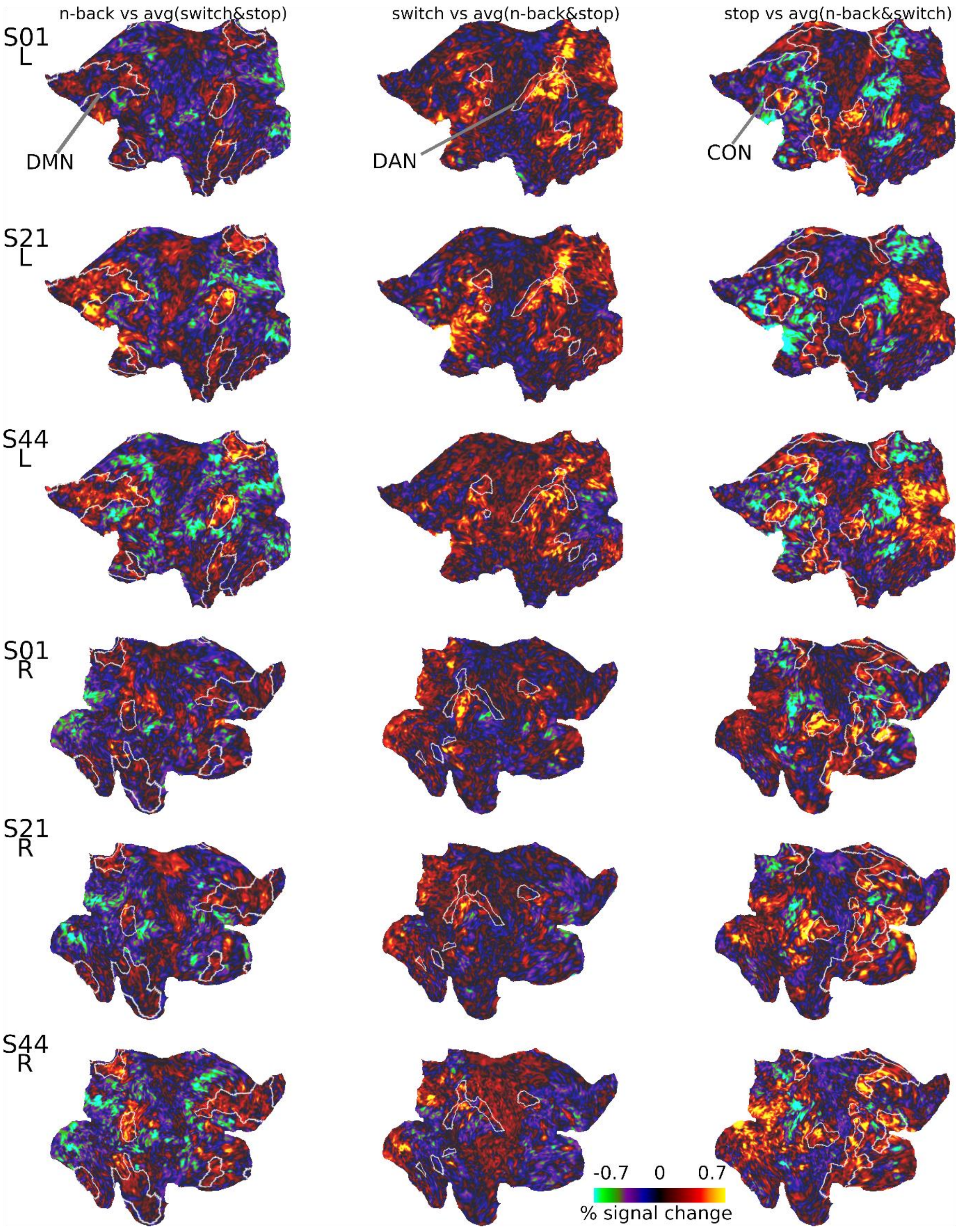

Supplementary Figure 3. Three example subject activations of each executive task minus the average of the two other tasks. RSN borders are outlined in white. Data available at: http://balsa.wustl.edu/G5P2G

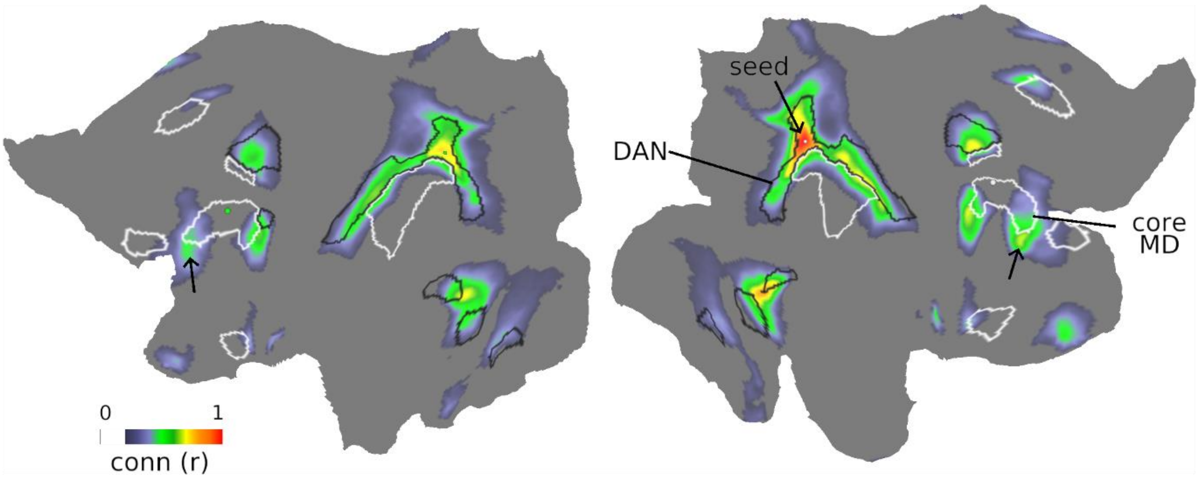

Supplementary Figure 4. Connectivity (Pearson's correlation) of a seed in DAN (black borders) shows connectivity to fine-grained regions ventral to the mid-frontal patch of core MD regions (white borders). Correlations are thresholded at 0.2. Correlations are average of the 210 validation HCP subjects (Glasser, Coalson, et al. 2016). Data available at: http://balsa.wustl.edu/qxPk9

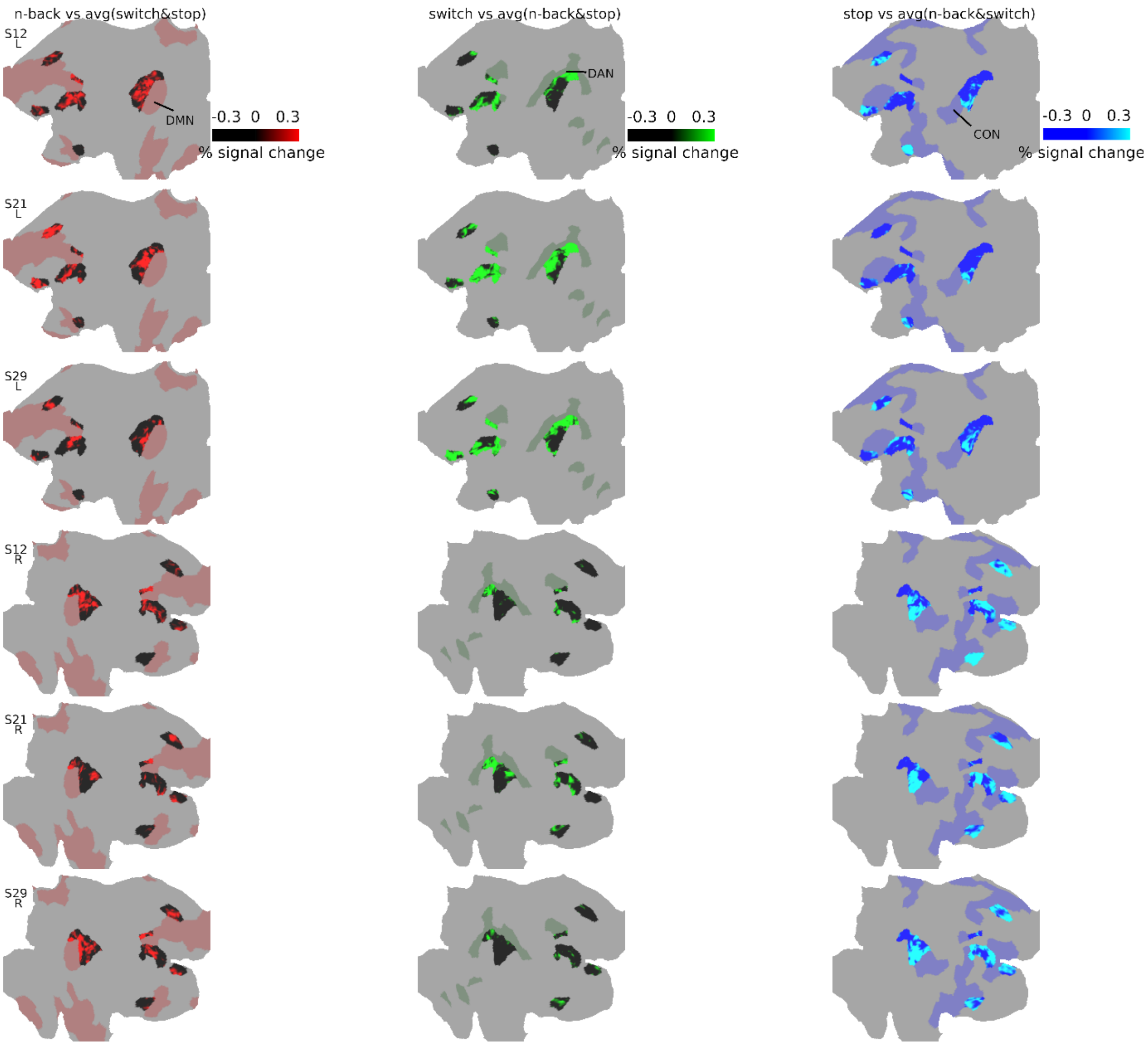

Supplementary Figure 5. Three example subject activations of each executive task minus the average of the two other tasks. First column shows n-back activations in red surrounded by DMN (faded red). Second column shows switch activations in bright green surrounded by DAN (faded green). Third column shows stop activations in cyan surrounded by CON (faded blue). Data available at: http://balsa.wustl.edu/L7qML

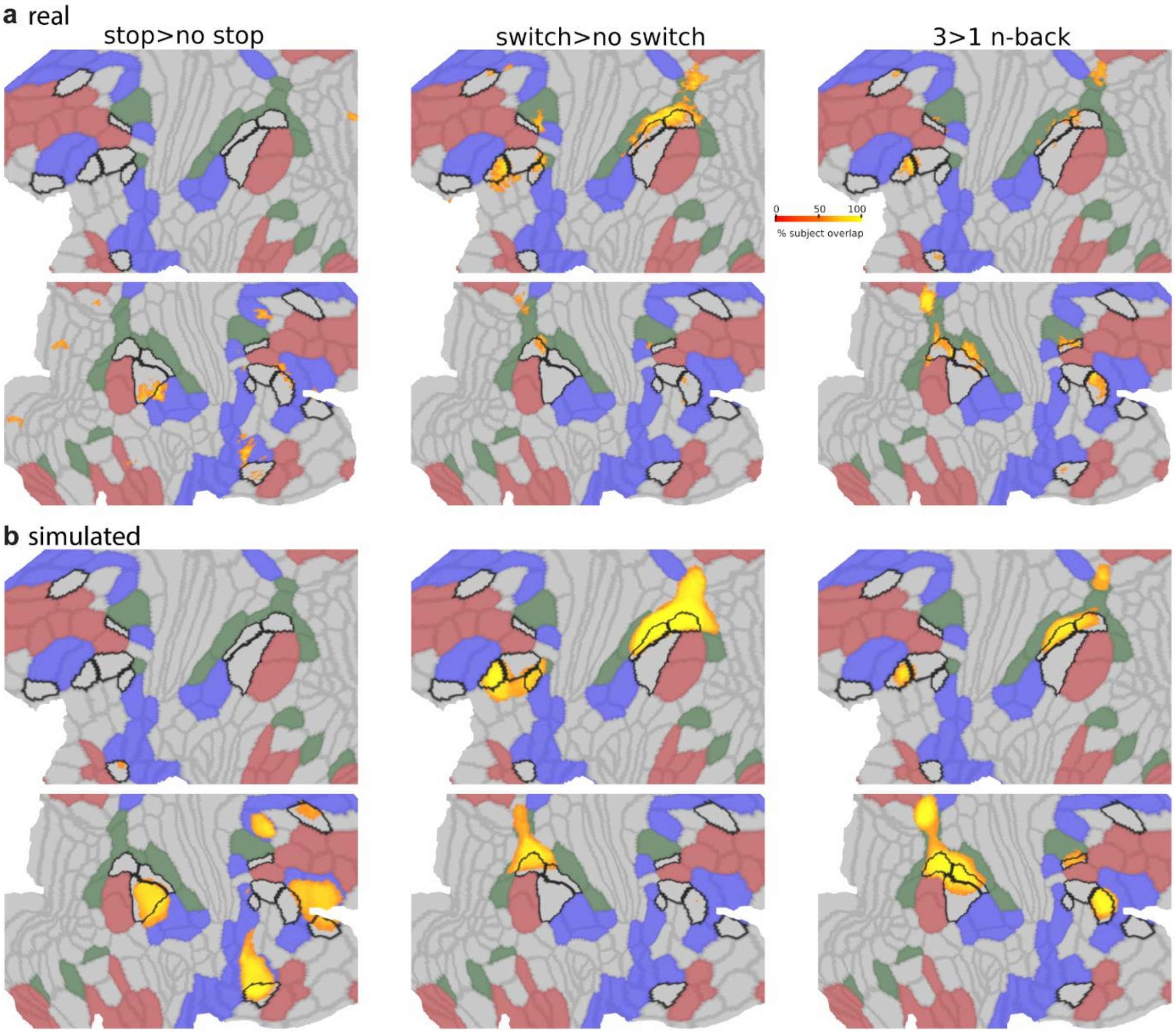

Supplementary Figure 6. Subject overlap maps for the top 5% vertices in real (top row) and 12 mm smoothed simulated data (bottom row). Data available at: http://balsa.wustl.edu/jNpZl

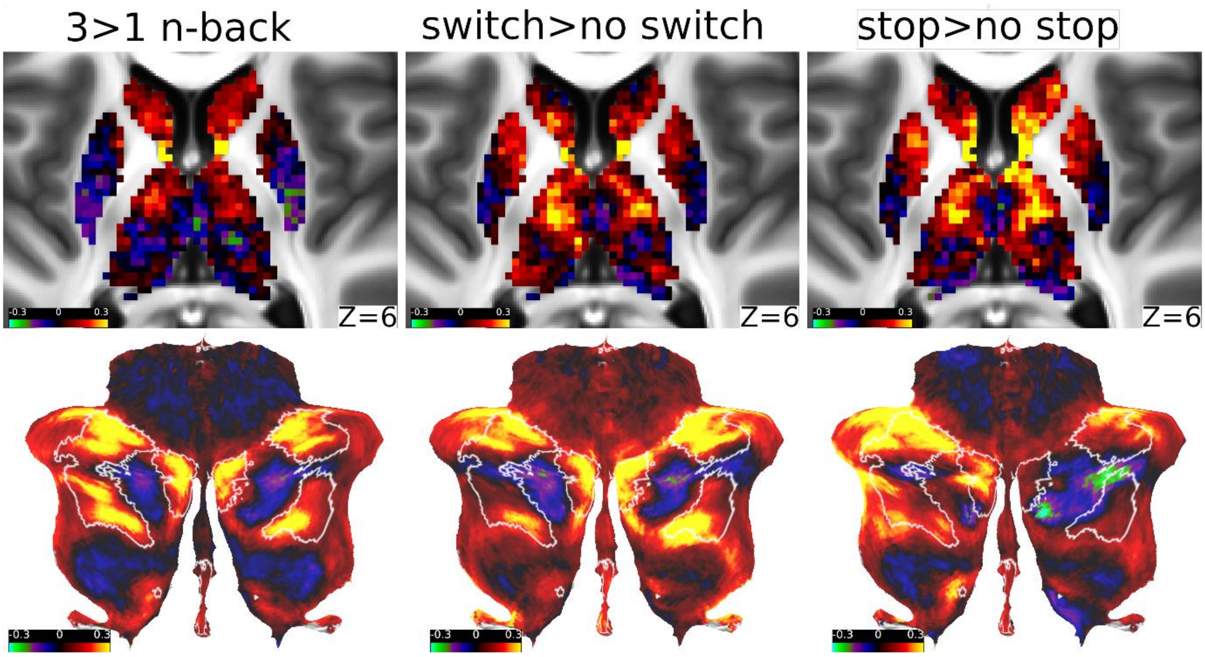

Supplementary Figure 7. Activations (percent signal change) for each executive contrast in an axial slice of the subcortex (top row) and a flat map of the cerebellum (bottom row). MD areas as defined in (Assem et al. 2020) are surrounded by white borders on the cerebellar surface. Data available at: http://balsa.wustl.edu/w8g71
